# Supplementary figures and images for: Correlation between the Cycle Threshold Values in Detection of Severe Fever with Thrombocytopenia Syndrome Virus Using PowerChekTM SFTSV Real-Time PCR Kit and Viral Load: Prognostic Implications
Source: Viruses. 2024 Apr 29;16(5):700. doi: 10.3390/v16050700 (PMC11125572; doi:10.3390/v16050700)

**Figure S1. Flowchart**

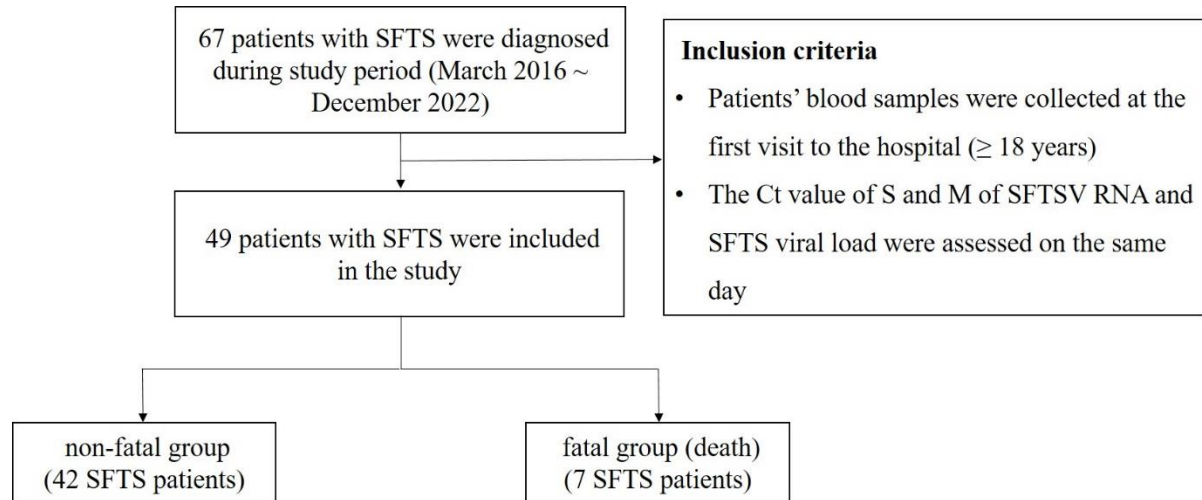

Supplement: Supplementary file 1 [file viruses-16-00700-s001.zip › viruses-2967642-supplementary.pdf]
